# Assessing the state of consciousness for individual patients using complex, statistical stimuli

**DOI:** 10.1016/j.nicl.2020.102471

**Published:** 2020-10-20

**Authors:** U. Górska, A. Rupp, T. Celikel, B. Englitz

**Affiliations:** a Computational Neuroscience Laboratory, Department of Neurophysiology, Donders Institute, Radboud University Nijmegen, The Netherlands; b Psychophysiology Laboratory, Institute of Psychology, Jagiellonian University, Krakow, Poland; c Smoluchowski Institute of Physics, Jagiellonian University, Krakow, Poland; d Section of Biomagnetism, Department of Neurology, University of Heidelberg, Heidelberg, Germany

**Keywords:** Disorders of Consciousness, EEG, Auditory, Textures

## Abstract

Patients with prolonged disorders of consciousness (PDOC) are often unable to communicate their state of consciousness. Determining the latter is essential for the patient's care and prospects of recovery. Auditory stimulation in combination with neural recordings is a promising technique towards an objective assessment of conscious awareness. Here, we investigated the potential of complex, acoustic stimuli to elicit EEG responses suitable for classifying multiple subject groups, from unconscious to responding.

We presented naturalistic auditory textures with unexpectedly changing statistics to human listeners. Awake, active listeners were asked to indicate the change by button press, while all other groups (awake passive, asleep, minimally conscious state (MCS), and unresponsive wakefulness syndrome (UWS)) listened passively. We quantified the evoked potential at stimulus onset and change in stimulus statistics, as well as the complexity of neural response during the change of stimulus statistics.

On the group level, onset and change potentials classified patients and healthy controls successfully but failed to differentiate between the UWS and MCS groups. Conversely, the Lempel-Ziv complexity of the scalp-level potential allowed reliable differentiation between UWS and MCS even for individual subjects, when compared with the clinical assessment aligned to the EEG measurements. The accuracy appears to improve further when taking the latest available clinical diagnosis into account.

In summary, EEG signal complexity during onset and changes in complex acoustic stimuli provides an objective criterion for distinguishing states of consciousness in clinical patients. These results suggest EEG-recordings as a cost-effective tool to choose appropriate treatments for non-responsive PDOC patients.

## Highlights

• Disorders of consciousness require objective measures of consciousness assessment.
• Complex, acoustic stimulation across 5 groups with different states of consciousness.
• Evoked responses alone are not sufficient to determine the states of consciousness.
• Dynamical complexity and evoked responses together correspond with clinical diagnosis.
• High accuracy when validated against behavioral consciousness scale.

## 1 Introduction

One of the most challenging clinical issues in patients with prolonged disorders of consciousness (PDOC) is to reliably estimate their residual, conscious perception of the environment. Vegetative state (VS; recently termed unresponsive wakefulness syndrome, UWS; (Laureys et al., 2010)) patients are believed to retain basic reflexes or sleep-wake cycles while remaining entirely unaware of self and environment (Monti et al., 2010). On the contrary, minimally conscious state (MCS) patients seem to preserve residual cortical functioning and display clear but inconsistent signs of awareness (Giacino and Schiff, 2009). Upon emergence from a minimally conscious state (EMCS) patients recover functional communication, although they often remain cognitively impaired (Di Perri et al., 2016). Several active (e.g. Cruse et al., 2011) and passive (e.g. King et al., 2013b) neuroimaging paradigms have suggested that some patients clinically classified as UWS can reveal signs of awareness and volitional control which argue that these patients should actually be classified as MCS, EMCS or locked-in syndrome (LIS). Considering that selection and administration of the appropriate rehabilitation programs necessarily require determination of the consciousness state, objective quantitative classification methods will facilitate PDOC treatment.

Following severe brain lesions that lead to PDOC states, it was suggested that the auditory system is less likely to be damaged in comparison with other parts of the brain (Kotchoubey et al., 2015). Moreover, audition was recently suggested to be particularly sensitive to fluctuations in the state of consciousness (Boly et al., 2004, Demertzi et al., 2014, Schiff et al., 2005). The results of a number of auditory studies that attempted to assess conscious processing with specific neural signatures in PDOC patients, using simple sounds (e.g. Binder et al., 2017), complex sound sequences (with P3b, e.g. Faugeras et al., 2012); but criticized by Tzovara et al., 2015), familiar sounds (Heine et al., 2015, Perrin et al., 2015), or speech (with N400, e.g. Steppacher et al., 2013) although (Rohaut and Naccache, 2017) presented it as a marker of early stage unconscious processing (Steppacher et al., 2013) remain partially inconsistent, specifically it remains unclear to what extent certain event-related responses co-exist with presence vs. absence of consciousness.

In the present study, we build on previous studies by (1) investigating a wide range of subject groups, from healthy responding subjects to unconscious patients, and (2) presenting common naturalistic auditory textures, i.e. complex sounds whose stimulus statistics change at a random time during stimulus presentation. Detecting these changes is a challenging, real-world task: in natural sounds, changes may occur unexpectedly, signalling potential dangers; consider for example listening to a busy street and discerning a car that turns towards you. Detecting these changes requires listeners to be aware of the recent acoustic statistics. In normal hearing subjects, we recently demonstrated (Boubenec et al., 2017) that the change in statistics leads to a characteristic centroparietal positivity (CPP, O’Connell et al., 2012). The CPP slope and size scales with the amount of evidence, suggesting that it reflects an underlying process of evidence integration (Boubenec et al., 2017, Kelly and O’Connell, 2013). Further, we found the CPP to depend on the level of processing, comparing between active, passive-aware (for subjects which had completed the active part before), and passive-naive (i.e. listeners for which the sounds were new, Górska et al., 2018). The fact that the CPP was still detectable even in passive-naive subjects encouraged us to use natural textures in order to assess the state of consciousness in a group of PDOC patients.

We find that the parietal signal, while prominent during waking, vanished during deep NREM sleep (first cycles during the night) and was not significantly present in the UWS and MCS patient groups. As an alternative analysis, we assessed the dynamical complexity of the neural response at the transitions of stimulus statistics using the Perturbational Complexity Index (PCI) measure, based on Lempel-Ziv complexity. We here refer to it as PCIa (audition) to indicate that the perturbation was an auditory stimulation. PCIa can simultaneously quantify integration and differentiation in the nervous system (Bodart et al., 2017, Casali et al., 2013, Casarotto et al., 2016) and thus has been proposed to estimate the state of consciousness irrespective of other related processes (Tononi et al., 2016). The results showed that PCIa can distinguish purportedly conscious (Responding, Non-Responding, MCS patients) from purportedly unconscious (NREM sleep, UWS patients), even on a single subject basis. We therefore propose that auditory textures in combination with complexity analysis provide a promising avenue for an objective assessment of the global state of consciousness even in passive PDOC patients.

## 2 Materials and methods

### 2.1 Subject groups and PDOC assessment

Multiple groups of PDOC patients and healthy volunteers participated in this study. All experiments were performed in accordance with the directives of the Helsinki Declaration (1975, revised 2000) and were approved by the Local Review Board of the Institute of Psychology, Jagiellonian University. Before participants were included in the study, healthy participants signed a written consent form, and informed consent was obtained from the legal surrogates of the PDOC patients.

The initial PDOC group consisted of 31 patients, however, some were excluded according to the three criteria of data quality. Specifically, (1) if the EEG amplitude exceeded +/- 200 μV in more than half of the trials, or (2) we observed specific movements artifacts e.g. teeth grinding, repeated jaw clamping, or (3) it was evident from the behavioural notes that a patient was sleeping for more than a half of the recording, subjects were excluded from further analysis. The latter was evaluated on the basis of the observable body reactions during the experiment. Based on these criteria seven subjects had to be excluded, leading to a final sample of 24 PDOC patients included in the subsequent analysis (mean age 40.43, sd: 14.38, 8 females).

These patients were behaviorally diagnosed on the basis of the Polish adaptation of the Coma Recovery Scale - Revised (CRS-R, Binder et al., 2018, Kalmar and Giacino, 2005*)*) as either UWS/VS (12 patients) or MCS and EMCS (5 and 7 i.e. 12 patients examined together)*,* see Table 1 for detailed information about the patients i.e. sex, age, etiology, time after the injury, CRS-R, the time between CRS-R and EEG. The CRS-R is a behavioural scoring tool consisting of six subscales that address auditory, visual, motor, oromotor, communication and arousal functions. It includes 23 items, hierarchically arranged in each subscale; starting from the lowest rate that represents reflexive responses, up to the highest rate representing cognitively mediated behaviours. CRS-R explicitly incorporates diagnostic criteria for UWS/VS (MSTF, 1994) and MCS (Giacino et al., 2002) and thus it received the strongest recommendation for differential diagnosis compared with other scales (Gerrard et al., 2014). Additionally, before the experiments, (transiently evoked) otoacoustic emissions were assessed for each DOC patient using an OtoRead™ device (Interacoustics, Middelfart, DK). This measurement employs a TEOAE protocol and only patients with SNR > 4 dB at 3/6 frequencies were included in the experiment.

**Table.1:** Clinical and demographic characteristics of PDOC patients. Total CRS-R score was marked in bold.

Patient	Age	Sex	Etiology	Time since injury (months)	Time between EEG and CRS-R acquisition (days)	CRS-R subscales						Total CRS-R score	diagnosis	The latest CRS-R total score	The latest diagnosis	Time since original CRS-R (days)
						auditory	visual	motor	oromotor	communication	arousal					
UWS1	38	M	anoxia	27	7	2	1	2	0	0	2	**7**	UWS	7	UWS	–
UWS2	48	M	anoxia	97	6	1	1	0	0	0	1	**3**	UWS	3	UWS	1078
UWS3	40	M	anoxia	49	6	1	0	2	0	0	1	**4**	UWS	5	UWS	1077
UWS4	59	M	anoxia + additional	14	15	1	0	1	0	0	1	**3**	UWS	3	UWS	–
UWS5	28	M	anoxia + diabetes	6	6	1	0	1	0	0	1	**3**	UWS	2	UWS	59
UWS6	52	M	anoxia	25	0	1	1	1	1	0	2	**6**	UWS	6	UWS	–
UWS7	28	M	anoxia + diabetes	11	0	1	0	1	0	0	0	**2**	UWS	2	UWS	–
UWS8	25	F	trauma + diabetes	23	0	1	0	1	1	0	1	**4**	UWS	4	UWS	413
UWS9	63	F	anoxia	11	0	1	0	2	1	0	1	**5**	UWS	5	UWS	40
UWS10	28	F	trauma	23	0	1	0	1	0	0	1	**3**	UWS	9	MCS	460
UWS11	30	F	trauma	9	1	1	0	1	0	0	1	**3**	UWS	3	UWS	–
UWS12	60	M	anoxia	5	0	2	0	0	1	0	1	**4**	UWS	3	UWS	20
MCS1	28	F	anoxia	69	7	4	5	4	1	2	3	**20**	EMCS	22	EMCS	595
MCS2	37	F	stroke	5	6	4	5	4	1	1	2	**18**	EMCS	17	EMCS	238
MCS3	30	F	trauma	12	0	3	5	2	1	1	2	**14**	MCS	9	MCS	98
MCS4	38	F	stroke	4	1	4	5	4	1	2	3	**16**	EMCS	21	EMCS	118
MCS5	30	M	anoxia	15	1	2	3	3	1	0	3	**12**	MCS	18	MCS	340
MCS6	38	F	stroke	18	1	4	5	5	1	2	3	**20**	EMCS	20	EMCS	–
MCS7	49	F	stroke	17	0	1	3	2	1	1	1	**9**	MCS	6	UWS	497
MCS8	22	M	trauma	3	0	3	5	2	1	2	3	**16**	MCS	5	UWS	321
MCS9	55	M	trauma	14	1	4	5	4	1	2	3	**19**	EMCS	5	UWS	575
MCS10	30	M	trauma	24	0	3	5	2	1	1	1	**13**	MCS	13	MCS	–
MCS11	34	M	trauma	8	0	4	5	2	1	2	3	**17**	EMCS	13	MCS	451
MCS12	22	M	trauma	11	0	4	5	6	1	1	3	**20**	EMCS	20	EMCS	–

The control groups included 28 normal hearing healthy volunteers, who all declared no substance abuse, were medication-free and did not report any neurological disorders. The first group (referred to as Awake Reporting below) consisted of 12 subjects (mean age 24.6 SD: 3.8, 8 females), the second group (Awake Passive) included 16 subjects, but only 12 of them (mean age 26.6 SD: 6.03, 6 females) were considered for further analysis due to the extensive noise recorded in at least one of the EEG sessions together with the prominent low-frequency activity e.g. extensive alpha evaluated from the occipital electrodes. The third group (Asleep NREM) was formed from subsets of the above groups, totalling 17 subjects, while 15 of them were analysed (mean age 25.2 SD: 4.2, 10 females; 12 subjects from the first and 4 from the second group). One subject was excluded because of the insufficient number of trials during NREM sleep and another due to extensive noise, which resulted in the rejection of more than ⅔ of trials based on the noise criterion, i.e. the amplitude larger than +/- 200 μV.

### 2.2 Stimulus design

A set of complex sounds, so-called naturalistic auditory textures (McDermott and Simoncelli, 2011), was presented to each subject. The set was drawn from a stimulus set used previously in healthy subjects (Górska et al., 2018), reduced here to account for the more limited, recording time available when working with (PDOC) patients. We provide here a brief description of the stimulus design, for a complete description see (Górska et al., 2018).

**Fig. 1:**
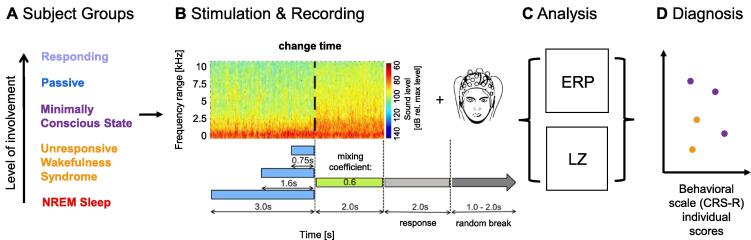
Using change detection in complex, statistical sounds for individual diagnosis of the state of consciousness. A We investigated EEG responses to acoustic stimulation in five subject groups with (presumably) differing levels of involvement. B The sounds were naturalistic textures - i.e. statistically defined sounds - which changed their statistics at unexpected times (depicted: 3 s). The changed statistics were a 60/40% mixture between the first sound and a second sound with different statistics. In the Responding group, listeners reported detecting a change in the sound via button press after the stimulus (see: response period). In the other groups (Passive, Minimally Conscious State (MCS), Unresponsive Wakefulness Syndrome (UWS), NREM Sleep), subjects passively listened to the same stimuli. Sounds were provided via headphones while simultaneously recording whole-head EEG signals from 64 channels. C We analysed the neural responses after stimulus onset and after the change in sound statistics via the evoked ERPs. Further we assessed the dynamical complexity using the Perturbational Complexity Index - auditory (PCIa). D The outcomes of both ERP and PCIa measures are finally compared on a single subject level with the behavioral diagnosis (CRS-S scale) to evaluate the potential for diagnosis.

Each sound was composed of a sequence of two auditory textures (see Fig. 1 for illustration). The first auditory texture followed the statistics of a single natural sound (sound of rain or bubbling in water), while the second texture was a linear mixture between the statistics of two natural sounds, one of which was the same as the first texture. This design allowed us to adjust the difficulty of the task. For the present set of subjects, the new texture contributed 60% to the second texture, while the first, baseline texture contributed only 40%. All textures were created using an openly available toolbox (McDermott and Simoncelli, 2011).

The transition between the sounds occurred at a pseudorandom time (either at 0.75 s, 1.6 s or 3 s), consisted of a linear mixing between the sounds over a duration of 10 ms with a sigmoidal profile over time (see Górska et al., 2018 for details). After the transition, each sound continued for 2 s. Every sound repetition was followed by an interstimulus interval (silence) of random length drawn from 1 to 2 s. Sham trials of matched lengths were included (50%), in which the first texture simply continued for the entire trial duration. To reduce the overall time of auditory stimulation in PDOC patients’ group, only one length of the sham type of trials (CT = 3 s) was used.

The natural textures used in the present experiment were based on two textures: the sound of rain and bubbles in water (see Fig. 1 for illustration). This choice was motivated by their level of similarity, i.e. the distinction between the two was neither trivial nor too difficult, as well as the fact that they both fulfilled the criteria of being spectrally and temporally broad and dense. We used multiple (i.e. 10) samples of each sound, which were matched in statistics, but differed in spectrotemporal details. In this way, a given statistic is not recognizable by a particular and static realization of the fine structure. The level of the stimulus was set to 70 dB SPL, which remained unchanged at and throughout the transition. Onsets and offsets were gated with a 10 ms sinusoidal function to reduce spectral broadening at the beginning and end of the sound.

## 3 Experimental procedure

The auditory textures were presented to awake and asleep subjects in separate experimental sessions. The complete dataset - EEG recording and CRS-R assessment - from each individual PDOC patient was registered during one of the five visits to the care centre in Torun, Poland.

### 3.1 Awake sessions

During awake sessions, control subjects were seated in an air-conditioned, soundproof chamber facing a monitor. After the procedure was briefly explained electrodes were attached to the scalp. The AEP recording was preceded by 2 min of resting-state registration. Subjects were instructed to maintain visual fixation on a white cross displayed in the middle of the screen. It was visible throughout the entire experiment for the Passive condition, while in the Responding condition it was temporarily removed after each stimulus to allow subjects to respond and blink if necessary.

The Awake Reporting group performed an active task in which they were asked to press a button after the offset of the sound if they detected the change in statistics (single response task). In each trial, the sequence was as follows: presentation of the sound texture, display of the question “Have you noticed a change?” on the screen, a 2-s response window to press the button, and then a silent period of randomized duration (to reduce the influence of expectation for the following stimulus onset). The response window was delayed to the moment after the sound to avoid motor response contamination of the EEG signal. No feedback was provided to the listeners regarding their correct/incorrect choice during the experiment. The procedure was composed of one session with 480 stimuli (i.e. 80 × 6 stimuli, 3 with and 3 without a change) and lasted ~ 65 min. The sequence of trials was randomized independently for each subject. The contamination due to blink artifacts was reduced by visually instructing (text message on the screen) to blink only during the between-trial silence.

The Awake Passive group was instructed to passively listen to the changing sounds while keeping eyes fixated on the cross displayed on the middle of the screen and trying not to think about anything in particular. The procedure consisted of 4 blocks with 60 trials each, for a total of 240 trials. Subjects were also asked to try not to blink during the sound presentation. After each block subjects were presented with a short questionnaire that consisted of three following questions: (1) ‘Did you succeed in not focusing on anything in particular?’, (2) ‘How much have you focused on changing sounds?’, (3) ‘To what extent your attention was directed to other external/ internal inputs?’ and they were requested to provide an answer in the Likert scale, i.e. from 1 (‘not at all’), to 5 (‘very much’). Results are not presented here, since this information was not available for all groups.

### 3.2 Asleep session

The asleep session was conducted in the same soundproof chamber, however, control subjects laid horizontally on a camp bed with electrodes attached and their head placed on a pillow. In order to maintain the regular sleep-wake cycle of the subjects, the experimental session started at about 23 h and lasted for 2–3 h (depending on the time it took subjects to fall asleep). The presentation of auditory stimulation commenced as soon as the experimenter recognized in the EEG signal the components specific for slow-wave sleep (i.e. N2 or N3 sleep phases). In these periods most of the stimuli are thought to be only processed automatically at lower levels and do not seem to be perceived consciously (Massimini et al., 2009, Strauss et al., 2015) as subjects do not usually recollect dreaming upon awakening (Gerrard et al., 2014, Massimini et al., 2005, Tononi and Massimini, 2008). The actual sleep phase was evaluated post-hoc during data-analysis by visual inspection of characteristic components i.e. sleep spindles or K-complexes (Berry et al., 2012). Otherwise, stimulus parameters were identical to those used in awake subjects and 240 trials (40 of each change and no-change stimuli) were presented.

### 3.3 PDOC patients session

The DOC patients’ sessions were conducted in separate rooms in the Care Center in Toruń, Poland. Patients were either seated on a wheelchair or at their bed in sitting upright position. During the experimental procedure, there were up to two experimenters in the room. Each patient completed one session of 320 stimuli (80 × 4 stimuli; 3 with the change and only one sham trial). This lasted for ~ 44 min. In some cases, the procedure was paused in the middle, in order to check the subject’s condition, improve electrode contacts, and continued subsequently. All other details of the procedure were the same as for the awake session of control subjects.

### 3.4 Experimental setup

#### 3.4.1 Presentation of acoustic stimuli

For all experimental conditions, stimuli were prepared using Matlab, then converted to analog signals and presented directly via Sennheiser MX 475 intra-aural earphones. The experimental procedures were implemented using Presentation software (NeuroBehavioral Systems, USA).

#### 3.4.2 Registration of EEG signals

EEG signals were acquired with a 64-channel ActiveTwo amplifier system, with active electrodes arranged on the scalp according to the 10–20 system (BioSemi, Amsterdam, NL). Four additional electrodes were located in the external canthi of both eyes and above and below the right eye, and another two were placed on mastoids and recorded in parallel. In order to ensure compatibility with the 10–20 system, we used standardized Electro-Caps in three different sizes (Electro-Cap International Inc., Eaton, USA). EEG signals were sampled at 1024 Hz, without highpass filter but a low-pass anti-aliasing filter (5th order, cascaded integrator comb digital filter), which limits the effectively available frequency range to 0–200 Hz (see www.biosemi.com for more details).

### 3.5 Data analysis

The analysis of the EEG data was separated into two steps. First, pre-analysis was performed using Brain Vision Analyser 2 (BVA, Brain Products, Germany) and then all further steps were done with custom-written scripts in Matlab (The Mathworks, Natick), based on tools provided in the EEGLAB toolbox (Delorme and Makeig, 2004).

For preprocessing, excessively noisy electrode channels were determined by visual inspection and replaced using spherical spline interpolation of the voltage from surrounding electrodes (Perrin et al., 1989); order: 4, degree: 10, lambda: 1E-05, BVA). In cases of excessive line noise, notch filtering at 50 Hz was applied (*Responding*: 1; *Passive:* 8; *Asleep NREM:* 3; *UWS*: 6, *MCS*: 6). The Ocular Correction ICA module implemented in BVA was applied to a subset of subjects from each group if substantial ocular artifacts were present (*Responding:* 3; *Passive:* 5; UWS: 5; *MCS:* 6).

In Matlab, the data were downsampled for further analysis to 128 Hz and re-referenced to the common average. Then, signals were high-pass filtered (15th order Chebyshev filter, using the Matlab function *cheby2*) at a conservative level of 0.3 Hz and low-pass filtered (4th order Butterworth filter, using the Matlab function *butter*) at 30 Hz. Epochs were extracted for each stimulus condition, which spanned the interval from 500 ms before stimulus onset to 1500 ms after stimulus offset. Next, epochs that contained artifacts exceeding +/- 200 μV were rejected. The remaining ERP data were baseline-corrected to the median voltage in each epoch at [150–400]ms window preceding the stimulus onset or change, respectively.

Data acquired during NREM sleep required additional preprocessing steps for offline selection of N2 and N3 phases epochs. This was performed in BVA by visual inspection of the data divided into 30 s epochs according to the American Academy of Sleep Medicine (AASM) criteria (Berry et al., 2012). Specifically, data classified as ‘N2′ or ‘N3′ were maintained, while ‘N1′ and ‘wakefulness’ were rejected from subsequent analysis. For the purpose of this checkup only, data were temporarily re-referenced, baseline corrected and filtered in the same way as for further analysis in Matlab.

### 3.6 EEG channel selection for analysis

We performed nonparametric permutation-based statistical analysis to identify electrodes that were significantly activated by the stimulus onset for each subject. The analysis was based on (Maris and Oostenveld, 2007), using the implementation in the FieldTrip package (ft_timelockstatistics, using 1000 randomizations). Specifically, we compared the silent prestimulus period with the *peri*-stimulus period of equal length (0.3 s), which provided joint sets of electrodes and time-periods of significant activation following the stimulus onset. The advantage of this method is that it identifies clusters evolving over time and channels, thus allowing to compare significant onset activations without predefining when and where they might occur, which is particularly important in the case of PDOC patients. This analysis was performed individually for all subjects of all groups. We limited the set of admissible electrodes to a broad set of central electrodes (comprising 15 electrodes: 'FC1′, 'FCz', ‘FC2′, 'FC3′, 'FC4′, 'C4′, 'C2′, 'Cz', 'C1′, 'C3′, 'CP3′, 'CP1′, 'CPz', 'CP4′, 'CP2′). For the P2 analysis the maximum of the evoked potential was taken within a widened time-range [0.1–0.3 s after stimulus onset].

*For non-patient groups*, the significantly and positively activated electrodes intersected across practically all subjects in the ‘FCz’, ‘FC1′, ‘FC2′ set. This set exhibited stronger significance and on average larger potentials than the classically used central set (‘Cz’, ‘C1′, ‘C2′), which was also significantly activated in all awake subjects (12/12). For non-patients, we therefore used this consistent set of significant electrodes (‘FCz’,’FC1′,’FC2′) for the P2 comparison.

*For the two patient groups* the choice was more complicated, since several subjects showed no significant P2 responses when using the same set of electrodes (UWS patients: 7/12, MCS patients: 3/12) in the first 300 ms after sound onset. In order to have a consistent criterion for choosing electrodes across subjects, we chose a set of electrodes of equal number as for the non-patients (3) which had the lowest p-values, independent of whether they fell below a significance threshold.

The selected electrodes for individual subjects including their p-values are reported in Supplementary Table 1 for all subjects and patients.

### 3.7 Complexity analysis

Disorders of consciousness may affect the diversity of brain responses and recent research has suggested the use of complexity measures to determine the state of consciousness (e.g. Casali et al., 2013, King et al., 2013a, King et al., 2013c). Accordingly we estimated the dynamical complexity of the EEG responses using the Lempel-Ziv compression algorithm (Wu et al., 2011). In a nutshell, it estimates the number of distinct sequences required to represent the neural activity reflected in the EEG. Following the implementation of (Schartner et al., 2015), we first transformed the data by downsampling to 64 Hz, and binarized it around a channel- and trial-specific threshold, computed as the average amplitude of the signal's Hilbert-transform. The resulting binary signal *S* was passed to an existing implementation of the Lempel-Ziv complexity estimate (Thai, 2020), which returned the size of the dictionary, i.e. the number of unique binary words used to represent the overall data in a compressed form. The word count was then converted to LZ complexity by multiplying it with the expected number of words for a random string, log2(L(S))/(L(S)∗H(S)), where *L(S)* is the length of *S* and *H(S)* the entropy of the source, defined as

H(S)=-P(S(i)=1)log2(P(S(i)=1)-(1-P(S(i)=1))log2(1-P(S(i)=1))

This estimate was verified against random strings with different probabilities of 0′s and 1′s, where it gave the value of the perturbational complexity index - audition (PCIa, Casali et al., 2013) of 1.

Thus, less stereotypic responses received higher complexity values, which have been proposed to be indicative of conscious activity.

The complexity analysis was applied to the data from a [-0.5–1.5 s] window around the onset / change time and for the latter, only one stimulus condition with the longest change time (3 s) was selected. The clusters of 14 channels around onset and change were selected, respectively.

### 3.8 Linear discriminant classification of state of consciousness

**Fig. 2:**
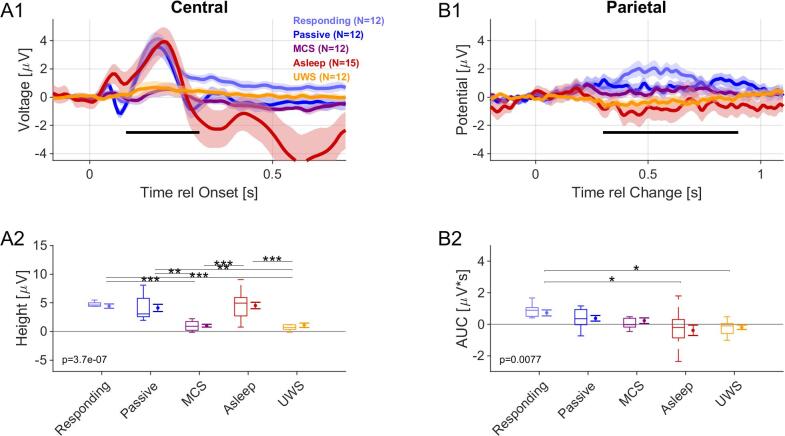
Onset and parietal ERPs distinguish several groups, but not conscious vs. unconscious. A1 An onset of an auditory stimulus creates an N1/P2 complex in a central location on the scalp (averaged electrodes: Cz, C1 and C2). Most saliently, healthy controls exhibited a clear P2 response. N1 responses were also discernible but only at a fraction of the P2′s size. In MCS and UWS patients the P2 response was strongly reduced and only remained discernibly>0 (see text for statistics) for MCS patients (see legend for color associations). A2 The P2 peak size was determined as the largest local peak inside a temporal window centered on the typical P2 latency (~225 ms), i.e. 100–350 ms. The median of the P2 height differed significantly across groups, and in particular, differentiated between healthy controls in all states of consciousness and the two patient groups (see figure and text for details). The p-value indicates a 1-way Kruskal-Wallis ANOVA across groups. Boxplots show the median with 25–75 percentiles, and the error bars indicate the mean +/- SEM. B1 The change-elicited response in the parietal region (averaged electrodes Pz, POz, P1, P2) reveal a slow, positive response peak, building up over a period of ~ 1 s (best visible for the Responding group). We computed the area under the curve (AUC) over the interval (0.2–1 s, black bar) to account for the variability of the response. B2 The AUC of the potential decreased generally from responding to UWS groups, however, significant differences were only found between the Responding and the Asleep/UWS groups (for statistical details see text). Plot elements as in A2.

**Fig. 3:**
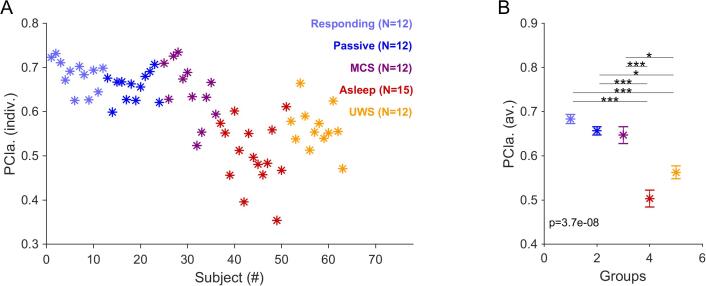
The complexity of the evoked EEG responses at change time decreases significantly with the level of consciousness. A Individual PCIa complexity scores computed for a [-0.25,1.25] s time window relative to change time across different consciousness groups/ While there is considerable within-group variation, the unconscious groups (Asleep (red) and VS patients (yellow)) lead to lower scores than the (presumably) conscious individuals (MCS (maroon), Passive (blue), Responding (light blue)). B The PCIa scores exhibited clear and significant differences between the unconscious and conscious groups (Kruskal-Wallis ANOVA: p < 0.00001, post-hoc tests between conscious and unconscious sets: all p < 0.05, Bonferroni corrected), while there were no significant differences within these sets (p > 0.05). The significance and effect size were much more pronounced than the same analysis performed on a corresponding window around stimulus onset (see Supplementary Fig. 3) and silent activity (see text). (For interpretation of the references to color in this figure legend, the reader is referred to the web version of this article.)

**Fig. 4:**
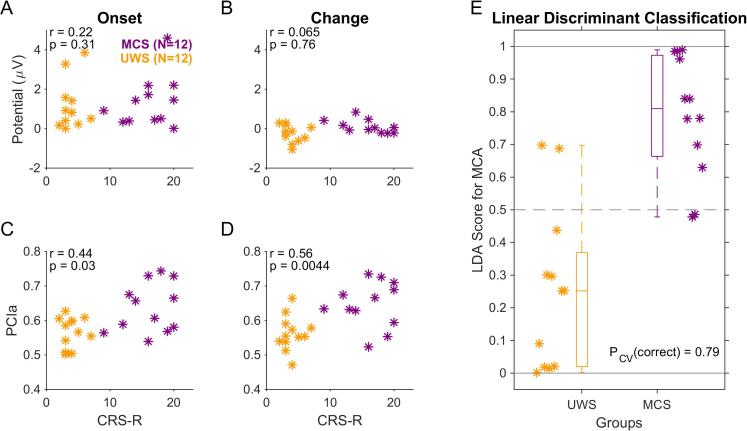
PCIa of the evoked EEG responses correlates best with the behaviorally assessed state of consciousness. A Peak potential values measured within the interval [0.2, 0.3] s related to sound onset from the central set of channels (P2 at stimulus onset) did not show a significant correlation with the total CRS-R score for individual PDOC patients. B The correlation of peak potential values measured within [0.6, 0.8] s related to change in acoustic texture from the parietal set of channels (CPP) and total CRS-R individual scores also remained insignificant. C PCIa computed over the interval [-0.5, 1.5] s relative to sound onset (from 14 channels, see Methods) correlate with total CRS-R individual scores, but only borderline significant. D PCIa computed over the interval [-0.5, 1.5] s relative to the change in statistics exhibited a strong correlation with the individual CRS-R scores of PDOC patients. Correlation is computed across both groups (MCS (maroon), UWS (dark yellow)) combined using Pearson's correlation (indicated in the top left corner of each plot with its significance). E Combining the P2 with the PCIa data via a linear discriminant analysis provides a correct classification rate of 79% (19/24 patients) on the basis of a cross-validated model estimate. (For interpretation of the references to color in this figure legend, the reader is referred to the web version of this article.)

The EEG measures (ERPs and PCIa estimates) were linked to the diagnosed state of consciousness using linear discriminant analysis (LDA) using the standard tools available in Matlab (fitcdiscr) and using cross-validation (cvshrink) to estimate robust performance. The input variables were the ERP measures (see Fig. 2) and the PCIa values (see Fig. 3) for both the medial/onset and the parietal/change potentials for each subject (for an overview see Fig. 4). The underlying model combines the input variables independently. The score (Fig. 4E) is computed by the *predict* function of the model returned by *fitcdiscr* in Matlab and indicates class membership between 0/1 with a threshold at 0.5 (since we here perform a binary classification).

### 3.9 General statistical analysis

If not specified otherwise, nonparametric tests were used. When data were normally distributed, we employed parametric alternatives to check that statistical conclusions were the same. One-way analysis of variance was computed using the Kruskal-Wallis test (Matlab function: kruskalwallis), followed by post-hoc testing using Wilcoxon Ranksum tests (Matlab function: multcompare) using Tukey's Honest Significant Difference correlation for multiple testing. Correlations were analysed using Spearman's rank-based method. Error bars represent ± 1 SEM (standard error of the mean). All statistical analyses were performed using the statistics toolbox in Matlab.

## 4 Results

We investigated the use of complex acoustic stimulation to distinguish between subject groups with varying levels of (task) involvement or consciousness state using a set of naturalistic auditory textures. Specifically, we compared five groups: *Responding*, *Passive* and *Asleep NREM* healthy controls, and two groups of patients with prolonged disorders of consciousness (PDOC), divided behaviourally (based on the CRS-R scale, see methods and Table 1) into the categories of *Minimally Conscious State (MCS)* and *Unresponsive Wakefulness Syndrome* (*UWS*, formerly referred to as *Vegetative State*, i.e. VS).

The statistical composition of the auditory stimuli changed only once per trial at a random time or remained unchanged (catch trials). Simultaneously, neural responses were collected using EEG. First, we compared the onset and change locked components of the neural response between groups using classical ERP analysis and then using the PCIa complexity measure (Casali et al., 2013, Ziv and Lempel, 1977).

### 4.1 The onset ERP only differentiates patients from healthy controls.

Onset activity for auditory stimulation is classically represented by a small, negative deflection at ~ 100 ms ('N1′) and a large, positive potential at 200–250 ms ('P2′), peaking at the central electrodes (Nie et al., 2014). Since the N1 component is relatively small for the present type of acoustic stimuli (Górska et al., 2018), we focused here on the P2 component (as in Górska et al., 2018). Additionally, in Asleep NREM condition, we detected a classical auditory multi-phasic response that consists of the N350, P450 and N550 components (Perrin et al., 2000). For the P2 analysis we used a set of 3 electrodes per subject, for healthy subjects, the commonly significant set [‘FCz’,‘FC1′,‘FC2′] and for patients an individual set of the 3 most significant electrodes selected from a broader set (see Methods).

The mean P2 potential differed significantly across all groups (1-way Kruskal-Wallis ANOVA, p≪0.0001, Fig. 2A1/2). For the healthy participants (i.e. Responding (light blue), Passive (dark blue), NREM Asleep (red), see Fig. 2A2) the P2 response represented a significant deflection (~2μV, significantly>0, p < 0.001 for all groups, Wilcoxon signed rank test, Tukey HSD corrected) and did not differ significantly between groups (p > 0.95, pairwise Wilcoxon ranksum tests). The largest local maximum within a window centered on the typical P2 interval (i.e. 150–300 ms after the response, indicated by the black bar in Fig. 2A1) was used to measure the individual P2 amplitudes. Thus, the mean of the individual values (Fig. 2A2) was typically larger than the peak of the corresponding grand-average curve (Fig. 2A1).

The median P2 response for MCS and UWS patients only exhibited a small positive excursion with an amplitude very close to 0 (Fig. 2A1). Both the MCS and UWS patients' responses were nonetheless significantly>0 (p < 0.05, Wilcoxon signed ranks test, Tukey HSD corrected). However, the P2 response did not differ between the two patient groups (p > 0.05, Wilcoxon Signed Ranks test for comparing medians, with Tukey HSD correction), although all healthy groups differed significantly from all patient groups (p < 0.01, Wilcoxon Signed Ranks test for comparing medians, with Tukey HSD correction).

For a subset of patients, onset P2 responses were visually detectable and differed significantly from 0 in single subject averages both for MCS (7/12, p < 0.05, nonparametric permutation-based statistical analysis, see Methods; Supplementary Table 1) and UWS (9/12, p < 0.05) groups (Individual P2 peaks were quantified as described above for the healthy controls; a representative, detectable onset response from patient groups is shown in Supplementary Fig. 1). The lack of significance for some patients could have been caused by a generally reduced level of cortical processing in these groups and background EEG with a dominance of ‘abnormal’ delta activity (Forgacs et al., 2014, Kotchoubey, 2005, Kotchoubey et al., 2005), which remains characteristic of patients with the most severe forms of structural injuries, usually most often in the UWS group (Schiff et al., 2014). However, a subset of the patients from the patient groups also exhibited more erratic motor activity, which contributes additional noise to the recordings, potentially masking the genuine ERPs. In particular, the set of UWS patients without a significant P2 response had a ~ 1.5 fold greater number of artifacts than even responding controls (0.8 vs. 0.5 / trials). Together, these factors could have reduced the P2 amplitude in these groups, rendering it closer to zero on average. The peak maxima of P2 (Responding: 0.19 ± 0.02 s; Passive: 0.19 ± 0.02 s; Asleep: 0.20 ± 0.02 s, MCS: 0.21 ± 0.03 s, UWS: 0.21 ± 0.04 s) did not differ significantly (p > 0.05, 1-way Kruskal Wallis ANOVA).

In summary, the onset ERP in response to the presented textural stimuli exhibited a strong difference between healthy controls and patients but did not allow to differentiate between patient groups.

### 4.2 The change-elicited ERP only differentiates responding controls from UWS and asleep subjects

In healthy subjects, the change in statistics for natural auditory textures was reflected by a late positive response in the parietal region (peak at ~ 650 ms post-change for the Responding group; see Fig. 2B1). Previous studies have shown that this response reflects properties of evidence integration required to make a decision on statistically defined stimuli (Boubenec et al., 2017, Górska et al., 2018). We therefore hypothesized that MCS and UWS patients may show some distinctive neural signature of stimulus integration.

Accordingly, we analysed a set of parietal electrodes (averaged Pz, POz, P1, and P2 channels), selected from the condition with the longest change time (3 s) as they represent the most prominent ERP at the scalp (Górska et al., 2018). In order to compensate for the increased variability in the patient groups, we used the area under the curve (AUC, computed as the sum of the potentials, divided by the integrated time, see Fig. 2B2, black bar) to quantify the strength of the parietal response (1-way Kruskal-Wallis ANOVA, p = 0.007, Fig. 2B2).

Similar to our previous results (Górska et al., 2018), we found the parietal response of the Passive group to be substantially lower on average (0.78 ± 0.19 vs. 0.38 ± 0.18 μV*s), although this comparison did not reach statistical significance for the present group. Significant differences were found between the Responding group and UWS (p = 0.03) and Asleep (p = 0.01) subject groups (2-group Wilcoxon rank sum test, Fig. 2B2; Table 2). Hence, evidence integration in complex stimuli - reflected in the parietal ERP size in Responding subjects (O’Connell et al., 2012, Boubenec et al., 2017, Górska et al., 2018) - differed between certain groups. Neither the MCS nor the UWS patient groups showed responses significantly>0 (p > 0.05, Wilcoxon Signed Ranks test).

**Table 2:** The series of comparisons in onset and parietal ERP responses between subsequent experimental groups with one-way Kruskal-Wallis ANOVA / t-tests. * was used for marking p < 0.05, ** for marking p < 0.001.

Comparison	Onset (central channels)		Change (parietal channels)	
	p - value	Cohen’s d	p - value	Cohen’s d
Responding vs. Passive	0.9872	0.178	0.896	0.548
Responding vs. MCS	0.000**	1.686	0.487	0.818
Responding vs. Asleep	1.0000	−0.057	0.001**	0.948
Responding vs. UWS	0.001**	1.573	0.030*	1.272
Passive vs. MCS	0.004**	1.376	0.953	0.297
Passive vs. Asleep	0.991	−0.192	0.173	0.693
Passive vs. UWS	0.006**	1.287	0.263	0.934
MCS vs. Asleep	0.000**	−1.440	0.585	0.561
MCS vs. UWS	0.999	−0.101	0.696	0.750
Asleep vs. UWS	0.000**	1.366	0.999	−0.191

### 4.3 Complexity of EEG activity following change differentiates across states of consciousness.

Recently, it has been suggested that the global state of consciousness is correlated with the dynamical complexity of neural activity recorded in EEG (e.g. Schartner et al., 2017, Thul et al., 2016). Specifically, methods to quantify the complexity of the brain response/activity were shown to carry diagnostic value for PDOC patients (Casali et al., 2013, Wu et al., 2011). The Lempel-Ziv measure estimates the set of unique activity patterns and has been proposed as a robust estimator of dynamical complexity of the ongoing EEG signal (see Methods for details). Here, we evaluated its ability to relate the response *evoked* by a complex stimulus with the global state of consciousness.

In the present dataset, we analysed a 1.5 s period around the sound onset or the change time [-0.25 to 1.25 s] using the index of complexity (PCIa) for each subject. Within-group averaged PCIa around both onset and change time reliably distinguished the purportedly conscious (Responding, Passive, MCS) from the purportedly unconscious groups (Asleep NREM, UWS). Individual PCIa values around change time are displayed in Fig. 3 (Fig. 3A and B, 1-way Kruskal-Wallis ANOVA overall groups, p≪0.0001, with p < 0.05 for all post-hoc Mann-Whitney tests between the purportedly conscious and unconscious subgroups). Individual PCIa of a similar window size around the onset time are shown in Supplementary Fig. 3.

We repeated the PCIa analysis for a duration-matched, silent period following the end of the stimulus. While the overall comparison across groups was still significant (p = 4.6e-5 for the central electrodes, p = 6.2e-6 for parietal electrodes), many post-hoc comparisons were non-significant, in particular the awake conditions could not be distinguished anymore from the UWS patients' condition. We therefore conclude that the neural response to the (complex) acoustic stimulation might be important for distinguishing the conscious and unconscious groups using the index of complexity. However, this is important to note that longer silence periods should be used in further research in order to allow for the complete return to baseline that would better match the resting-state condition.

Further, we tested how well the ERP size and PCIa correlated with behavioral indicators (CRS-R scale) and the derived clinical classification. For the P2 activity evoked by stimulus onset, we did not find a positive correlation with CRS-R (Pearson’s r = 0.22; p = 0.31; Fig. 4A), as well as it remained non-significant for the CPP activity following the change in stimulus statistics (r = 0.065; p = 0.76; Fig. 4B). Alternatively, PCIa at the stimulus onset and change correlated significantly with the CRS-R total score (r = 0.44; p = 0.03, Fig. 4C; r = 0.56, p = 0.0044, Fig. 4D, respectively).

Next, we combined the P2 with both PCIa outcomes using linear discriminant analysis in order to assess a combined estimate for the clinical classification of the state of consciousness. Estimates for individual subjects were created using leave-one-out cross-validation, and thus represent a lower bound for the actual model performance (Sahani and Linden, 2003). The estimates indicated a correct classification rate of 79%, corresponding to a ratio of 19/24 patients (Fig. 4E). Moreover, the LDA analysis on the same data, but with shuffled labels (and crossvalidation), had the expected random predictive success of 50%. Further, we related ERP and PCIa data with CRS-R subscales, revealing that Auditory, Visual, Motor and Communication subscales correlate significantly with PCIa at both onset and the change, and Arousal subscale also correlated with PCIa at change. The detailed correlations for each measure are presented in Supplementary Table 2. Additionally, we provided a comparison between all tested groups and conditions with the confusion matrix (Supplementary Fig. 4A), revealing that most confusions seemed to happen within, respectively, the purportedly conscious and unconscious groups, rather than across.

**Fig. 5:**
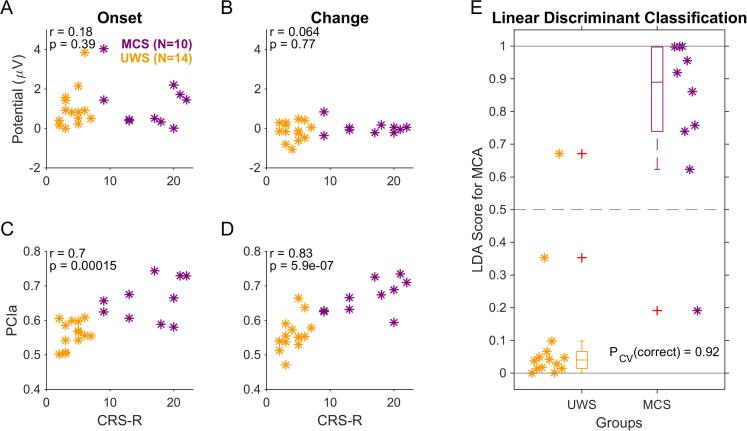
Consciousness state classification improves when comparing against the latest available clinical assessment. Layout and statistics as in Fig. 4. The correlations for the PCIa measures improved for both the medial (C) and parietal (D) electrodes. Most importantly, the classification of the state of consciousness became more accurate with only 2/24 patients misdiagnosed by the EEG-based LDA classifier (E).

For most of the patients, who continued to be treated in the Care Center, the behavioural assessment of CRS-R was repeated multiple times over the years of their stay (but not as a part of the present EEG study). Within this group, some patients tend to recover together with obtaining a higher CRS-R score, while some revealed a lower level of overall functioning and lower total scores than before. According to the CRS-R administration closest to EEG measurement and the latest CRS-R available (N = 16, 399 days on average, see Table 1), 4/16 changed diagnosis. Lastly, we explored how the classification on the basis of EEG recordings at a given point in time relates to the future classification of the subjects' state of consciousness. We therefore collected for each patient the latest available CRS-R score and diagnosis (see last three columns in Table 1) and repeated the analysis above (see Fig. 5). On the basis of the EEG recording, the classification accuracy improved to 92% (Fig. 5E, 22/24). Similarly, the correlations with CRS-R increased for the PCIa for both the medial and the parietal electrodes (Fig. 5C-D). The significance of separating all groups based on the PCIa around change time also improved on the group level (p = 7e-9, compared to p = 3.7e-8 from Fig. 3), together with less confusions (4 vs 10 cases) between purportedly conscious and unconscious groups (Supplementary Fig. 4B).

In summary, using both the ERP and the index of complexity of the neural responses around the onset of the statistical stimuli, we find a significant relationship with the global state of consciousness. However, PCIa at the change in statistics was a substantially better predictor for the state of consciousness than the ERPs after the change in stimulus statistics. A linear discriminant model of the P2 and PCIa measures provided the best performance with high classification accuracy. Overall, PCIa around the change in acoustic stimulus statistics is a good candidate for assessing the level of consciousness in non-responsive patients.

## 5 Discussion

In the present study, we tested the capability of using complex acoustic stimuli to reliably differentiate between various states of consciousness. Specifically, we investigated whether such a cost-effective and comparatively easy-to-use method could be clinically useful for distinguishing minimally conscious and unresponsiveness wakefulness syndrome patients. We found that a measure of dynamical complexity (perturbational complexity index - audition, PCIa) was comparable in distinguishing these conditions at both onset and change in statistics. Combining across these methods provided the best classification performance, which could distinguish the purportedly conscious/unconscious groups on the single subject level.

### 5.1 EEG measures for differentiating between states of consciousness

Objective measures for assessing the state of consciousness are important in the clinical assessment of patients with prolonged disorders of consciousness (PDOC). In particular, in PDOC patients the state of consciousness is often decoupled from measurable behavior, and might be distinct from responsiveness, hence, it is essential to develop objective indicators of consciousness based on neural responses in passive, no-report paradigms (as recently emphasized by Tsuchiya et al., 2015). Previous studies have suggested that the response of (primary) auditory cortex is preserved in PDOC patients (Laureys et al., 2004) and usually characterized by a cortical N1-P2 complex (Kotchoubey, 2005), which remains recognizable in about 80% of PDOC patients (Kotchoubey, 2005) and is more frequent in MCS than UWS patients (Kotchoubey, 2005, Perrin et al., 2006). The preserved response of the primary auditory cortex was even suggested as a useful predictor for recovery (Fischer et al., 1999).

In our study, evoked responses were less well correlated with behavioral scores of individual subjects than the PCIa measure of complexity (see below). For the complex stimulus used herein, the P2 onset component was pronounced (Górska et al., 2018): While prominent and conserved in size across the healthy controls groups, it could not reliably differentiate between MCS and UWS patients (Fig. 2), even on the population level. Although it tends to be more pronounced in the MCS group, and appeared indicative of UWS patients whose behavioural diagnosis later changed into MCS (see: Fig. 5).

Recent studies had debated the relation of the P3 activity (which appears quite related to centro-parietal positivity (CPP) activity; (Boubenec et al., 2017, Górska et al., 2018, Kelly and O’Connell, 2013, O’Connell et al., 2012) with consciousness: earlier studies (Bekinschtein et al., 2009) suggested that the P3 response reflects conscious awareness, while recent studies argued to dissociate it from conscious experience and consider it more as a complex novelty response (Koch et al., 2016) or the marker of responsiveness (Verleger, 2020). Presently, we find the CPP not to be suitable for distinguishing MCS and UWS patients. While it exhibited a reliable, slow positive response in Responding subjects, and progressively vanished in Passive and NREM sleep, it was insufficient to distinguish between the other groups (which - at least for the Passive group - may have been due to small group size and the smaller number of conditions used presently (Górska et al., 2018)). Previous studies suggest that the presence of P3 component in PDOC patient's group is usually limited to a small subset of cases in the tested group (e.g. in Faugeras et al., 2012): the global effect of P3b was found in 4/28 MCS and 2/24 UWS patients, see also (Chennu et al., 2013, Kempny et al., 2018, Real et al., 2016) for similar effects). Moreover, most of those research aimed to elicit P3 activity with an earlier maxima (e.g. 400–600 ms in response to own name, (Sergent et al., 2017), while the CPP observed in reaction for the change in natural auditory texture is relatively late component and its formation possibly engages higher order cortical areas i.e. medial parietal cortex and a late response in auditory cortex (Gorska et al., 2018). While the CPP size significantly differed across the five groups, it did not distinguish reliably between the different non-responding groups. Hence, the evidence integration process required for statistical change detection only appears to be strong in actively participating subjects (Del Cul et al., 2007, Górska et al., 2018, Sergent et al., 2005), which may limit the activation also in MCS patients, whose degree of participation cannot be confirmed. An interesting modification would be to introduce stimuli with similar complexity, but higher emotional value, which has been shown to lead to larger responses (Heine et al., 2015).

On the other hand, we found that signal complexity based on the Lempel-Ziv measure represents a reliable estimate of the state of consciousness, distinguishing Active, Passive and MCS groups from the supposedly unconscious (NREM Asleep, UWS) groups. Specifically, the estimation of PCIa around the change in complex stimulus statistics seems to reflect the state of consciousness even when some reliable ERP could not be derived, and, importantly, the comparison remains more pronounced when compared to the baseline periods following each stimulation. A link between dynamical complexity and the actual state of consciousness had already been demonstrated (Tononi and Edelman, 1998) and accordingly multiple complexity measures have been devised to classify these states (Bodart et al., 2017, Schartner et al., 2015, Thul et al., 2016). This series of studies demonstrated that the EEG response to transcranial magnetic stimulation (TMS) either differs between electrodes and spreads across the whole cortex (conscious states) or it remains more stereotypical across electrodes and local to the site of stimulation. This was demonstrated for multiple states of unconsciousness, including deep sleep (Massimini et al., 2005), anesthesia, (Sarasso et al., 2015), and brain injuries, (Rosanova et al., 2012, Casarotto et al., 2016). In a previous study (PCI; Casali et al., 2013), the perturbational complexity index in response to TMS also revealed relatively low values for unconscious states, similar to the present PCIa results for the Asleep and UWS groups.

Our results are consistent with the hypothesis that the perturbation used to measure signal complexity can also be introduced via sensory stimulation (Sitt et al., 2013). While PCI is believed to reflect complexity by simultaneously assessing integration and differentiation, PCIa could be related to a representation of a difference between the spectrotemporal composition before and after the change in statistics. However, this could also be reflected in a similar pattern of responses in a group of far spatially distributed channels, since the cortical channels receive strong inputs from subcortical structures. It has been shown that in NREM sleep, as well as UWS the cerebral cortex retains some reactivity (Gosseries et al., 2015), but remains blocked in a state of low complexity (Casarotto et al., 2016). Recently, it was proven that cortical activity in UWS patients exhibits a pathological tendency to fall into periods of silence during down states, preventing the buildup of any complex response upon receiving an input (Rosanova et al., 2018).

The complexity of neural activity has been shown previously to significantly discriminate between UWS and MCS patients, particularly for a set of electrodes over the parietal region (Sitt et al., 2014). This tendency corresponds to our findings where the discrimination between conscious and unconscious groups was lower to the onset as compared to the change response.

### 5.2 Limitations of using the CRS-R scale as a reference for classification

The behavioural assessment represents an indirect (mainly motor) measure of consciousness, and thus it may misclassify patients who are for example conscious but unable to move (Giacino et al., 2009); . The CRS-R scale is a trusted tool for assessing the state of consciousness (Schnakers et al., 2009), however, it is also not without limitations (Bodien et al., 2016).

CRS-R assessment of the same patient may change over longer periods, although typically it should remain stable over several days (Beukema et al., 2016, Schiff et al., 2007) and exhibits high test–retest reliability (Seel et al., 2010, La Porta et al., 2013). Due to technical limitations, we were not able to administer the CRS-R test on exactly the same day as the EEG measurement (average temporal separation: 2.7 days). Recently, multiple administration of CRS-R was recommended in order to reduce the level of ambiguity in assessing the clinical condition of patients (Wannez et al., 2017). Thus, its single assessment in the current study may have increased the measurement error in comparison to multiple assessments. As a consequence, it cannot be ruled out that the lower accuracy of CRS-R result might have weakened the estimates of the existing relationship between the clinical state of the patient and his or her response to auditory textures. This may have added variability to the relation between clinical assessment and the neural predictor variables, although the relation remained overall quite strong (Fig. 4D).

In the present study, we used clinical assessment (based on the CRS-R scores) as our reference for classification. While we find overall a good agreement of the ERP/PCIa-based linear discriminant classification with the CRS-R score estimated close in time to the EEG measurement (Fig. 4E), the classification improved when taking the follow-up CRS-R scores into account (Fig. 5E). This re-emphasizes the value of non-behavioral, brain-based measures of the state of consciousness.

### 5.3 General limitations and future improvements to the study design

In the present analysis, we focused on the properties of the evoked responses (ERPs), which seem to lack sensitivity in comparison with the estimation of dynamical complexity. However, single subject classification could benefit from a combination of these properties (Sergent et al., 2017, Sitt et al., 2014), or even train a general classifier to categorize the global state of consciousness based on the entire set of neural recordings from a subject.

Furthermore, we focused on EEG and acoustic stimulation as it is readily available, inexpensive and easy to administer in clinical settings. Future studies could investigate whether a combination of related techniques (e.g. TMS or fNIRS (functional near-infrared spectroscopy)) could improve classification performance or conversely, whether even simpler EEG systems with fewer electrodes could lead to comparable results, and thus allow the present analysis using much simpler setups. This is particularly important for recording sessions which have to be interrupted by clinical interventions, as is often the case in intensive care units.

Finally, since here we tested the number of groups in various states/levels of involvement, the number of subjects was more limited than in previous studies. In a follow-up study, we aim to increase the sample size of patients in particular, in order to divide them based on their etiology, while presently, there was a prevailing proportion of anoxic patients (11/18; see Table 1). This will also allow to validate the outcome of the classification on the new groups tested under the same conditions. Moreover, a larger sample size and increased recording time could allow the separation of all sleep stages in the healthy group or even the assessment of naturally disturbed sleep patterns in PDOC patients (Cologan et al., 2010, Malinowska et al., 2013, Pavlov et al., 2017, Wielek et al., 2018).

## 6 Conclusions

Recently, many algorithmic solutions have been developed to quantify the correlates of intact cognitive processes in PDOC patients. While we find basic ERPs to be insufficient for diagnosing individual patients, the combination of complex acoustic stimuli and dynamical complexity of the neural response appears promising for aiding the diagnosis of PDOC patients.

## CRediT authorship contribution statement

**U. Górska:** Conceptualization, Methodology, Formal analysis, Investigation, Writing - original draft, Writing - review & editing, Funding acquisition. **A. Rupp:** Formal analysis, Writing - review & editing. **T. Celikel:** Writing - review & editing, Resources. **B. Englitz:** Conceptualization, Methodology, Software, Formal analysis, Resources, Writing - review & editing, Supervision.

## Data availability

All raw data required to reproduce all analyses and figures are uploaded onto the Donders Data Repository and can be found at: https://data.donders.ru.nl/doc/dua/RU-DI-HD-1.0.html?0.

## Declaration of Competing Interest

The authors declare that they have no known competing financial interests or personal relationships that could have appeared to influence the work reported in this paper.
