# Supplementary material for: Assessing the state of consciousness for individual patients using complex, statistical stimuli
Source: Neuroimage Clin. 2020 Oct 20;29:102471. doi: 10.1016/j.nicl.2020.102471 (PMC7788231; doi:10.1016/j.nicl.2020.102471)
Supplement: Supplementary data 1 [file mmc1.docx]

**Supplementary Data:**

**Supplementary Table 1.** Channels and latencies selected based on nonparametric permutation-based statistical analysis of individual ERP responses at onset (P2) in acoustic texture, respectively. * indicates p<0.05.

| **Group + subject no** | **Selected channels** | **channels p-values** | **Significant Latency within [0.1 - 0.3] seconds** |
| --- | --- | --- | --- |
| Resp1 | FCz, FC1, FC2 | 0.001* | [0.11 - 0.25] |
| Resp2 | FCz, FC1, FC2 | 0.001* | [0.11 - 0.30] |
| Resp3 | FCz, FC1, FC2 | 0.001* | [0.14 - 0.30] |
| Resp4 | FCz, FC1, FC2 | 0.001* | [0.13 - 0.30] |
| Resp5 | FCz, FC1, FC2 | 0.001* | [0.12 - 0.24] |
| Resp6 | FCz, FC1, FC2 | 0.001* | [0.12 - 0.23] |
| Resp7 | FCz, FC1, FC2 | 0.001* | [0.12 - 0.24] |
| Resp8 | FCz, FC1, FC2 | 0.001* | [0.16 - 0.25] |
| Resp9 | FCz, FC1, FC2 | 0.001* | [0.12 - 0.30] |
| Resp10 | FCz, FC1, FC2 | 0.001* | [0.12 - 0.23] |
| Resp11 | FCz, FC1, FC2 | 0.001* | [0.11 - 0.30] |
| Resp12 | FCz, FC1, FC2 | 0.001* | [0.11 - 0.16] |
| Passive1 | FCz, FC1, FC2 | 0.001* | [0.11 - 0.24] |
| Passive2 | FCz, FC1, FC2 | 0.001* | [0.21 - 0.27] |
| Passive3 | FCz, FC1, FC2 | 0.001* | [0.13 - 0.20] |
| Passive4 | FCz, FC1, FC2 | 0.001* | [0.16 - 0.21] |
| Passive5 | FCz, FC1, FC2 | 0.001* | [0.12 - 0.22] |
| Passive6 | FCz, FC1, FC2 | 0.001* | [0.18 - 0.23] |
| Passive7 | FCz, FC1, FC2 | 0.001* | [0.13 - 0.22] |
| Passive8 | FCz, FC1, FC2 | 0.001* | [0.17 - 0.23] |
| Passive9 | FCz, FC1, FC2 | 0.001* | [0.13 - 0.24] |
| Passive10 | FCz, FC1, FC2 | 0.001* | [0.12 - 0.23] |
| Passive11 | FCz, FC1, FC2 | 0.001* | [0.15 - 0.20] |
| Passive12 | FCz, FC1, FC2 | 0.003* | [0.14 - 0.24] |
| MCS1 | FC3, FC1, FCz | 0.030* | [0.15 - 0.17] |
| MCS2 | FCz, FC1, FC2 | 0.001* | [0.14 - 0.25] |
| MCS3 | FC3, FC1,CP2 | 0.020* | [0.27 - 0.30] |
| MCS4 | FCz, FC1, FC2 | 0.001* | [0.11 - 0.17] |
| MCS5 | CP2, Cz, CPz | 0.001* | [0.12 - 0.14] |
| MCS6 | FCz, FC1, FC4 | 0.001* | [0.18 - 0.20] |
| MCS7 | FC2, FCz, FC4 | 0.400 | [0.20 - 0.21] |
| MCS8 | FCz, Cz, C1 | 0.060 | [0.14 - 0.23] |
| MCS9 | FCz, FC1, FC2 | 1 | - |
| MCS10 | FC2, FC1, C1 | 0.155 | [0.18 - 0.21] |
| MCS11 | FCz, FC1, FC2 | 0.011* | [0.1 - 0.17] |
| MCS12 | Cz, C1, C2 | 0.060 | [0.18 - 0.21] |
| Asleep1 | FCz, FC1, FC2 | 0.001* | [0.16 - 0.24] |
| Asleep2 | FCz, FC1, FC2 | 0.001* | [0.10 - 0.21] |
| Asleep3 | FCz, FC1, FC2 | 1 | - |
| Asleep4 | FCz, FC1, FC2 | 0.001* | [0.16 - 0.21] |
| Asleep5 | FCz, FC1, FC2 | 0.006* | [0.20 - 0.24] |
| Asleep6 | FCz, FC1, FC2 | 0.001* | [0.14 - 0.23] |
| Asleep7 | FCz, FC1, FC2 | 0.001* | [0.17 - 0.24] |
| Asleep8 | FCz, FC1, FC2 | 0.001* | [0.1 - 0.15] |
| Asleep9 | FCz, FC1, FC2 | 0.001* | [0.19 - 0.23] |
| Asleep10 | FCz, FC1, FC2 | 0.001* | [0.17 - 0.23] |
| Asleep11 | FCz, FC1, FC2 | 0.001* | [0.14 - 0.26] |
| Asleep12 | FCz, FC1, FC2 | 0.001* | [0.12 - 0.27] |
| Asleep13 | FCz, FC1, FC2 | 0.020* | [0.17 - 0.24] |
| Asleep14 | FCz, FC1, FC2 | 0.001* | [0.16 - 0.22] |
| Asleep15 | FCz, FC1, FC2 | 0.001* | [0.16 - 0.22] |
| UWS1 | FCz, FC1, FC2 | 0.001* | [0.16 - 0.26] |
| UWS2 | FC1, FC2, FC3 | 0.001* | [0.14 - 0.30] |
| UWS3 | FCz, FC1, C1 | 0.035* | [0.21 - 0.22] |
| UWS4 | FCz, FC1, FC2 | 0.070 | [0.13 - 0.19] |
| UWS5 | CP4, FC3, FC1 | 1 | - |
| UWS6 | FCz, FC1, FC2 | 0.001* | [0.12 - 0.19] |
| UWS7 | FCz, FC1, FC2 | 0.009* | [0.20 - 0.24] |
| UWS8 | Cz, FCz, FC2 | 0.001* | [0.19 - 0.30] |
| UWS9 | FCz, FC1, Cz | 0.180 | [0.10- 0.11] |
| UWS10 | FCz, FC1, FC2 | 0.001* | [0.10 - .0.24] |
| UWS11 | FCz, FC1, FC2 | 0.021* | [0.14 - 0.19] |
| UWS12 | FCz, FC1, FC2 | 0.001* | [0.15 - 0.19] |

**Supplementary Table 2.** The set of correlations with ERP and PCIa measures at stimulus onset and change, respectively, and CRS-R subscales. * indicates p<0.05.

| **CRS-R subscale** | **ERP onset** | **ERP change** | **PCIa onset** | **PCIa change** |
| --- | --- | --- | --- | --- |
| Auditory | r=0.170; p=0.410 | r=0.078; p=0.720 | r=0.460; p=0.025* | r=0.510; p=0.011* |
| Visual | r=0.230;  p=0.280 | r=0.320; p=0.130 | r=0.480; p=0.018* | r=0.530; p=0.008* |
| Arousal | r=0.260; p=0.220 | r=0.240; p=0.260 | r=0.310; p=0.150 | r=0.430; p=0.036* |
| Motor | r=0.028;  p=0.900 | r=0.130; p=0.540 | r=0.340;  p=0.1 | r=0.540; p=0.006* |
| Oromotor | r=0.064; p=0.770 | r=-0.089; p=0.680 | r=0.370; p=0.075* | r=0.260; p=0.230* |
| Communication | r=0.220; p=0.031* | r=0.180; p=0.410 | r=0.470; p=0.021* | r=0.520; p=0.009* |


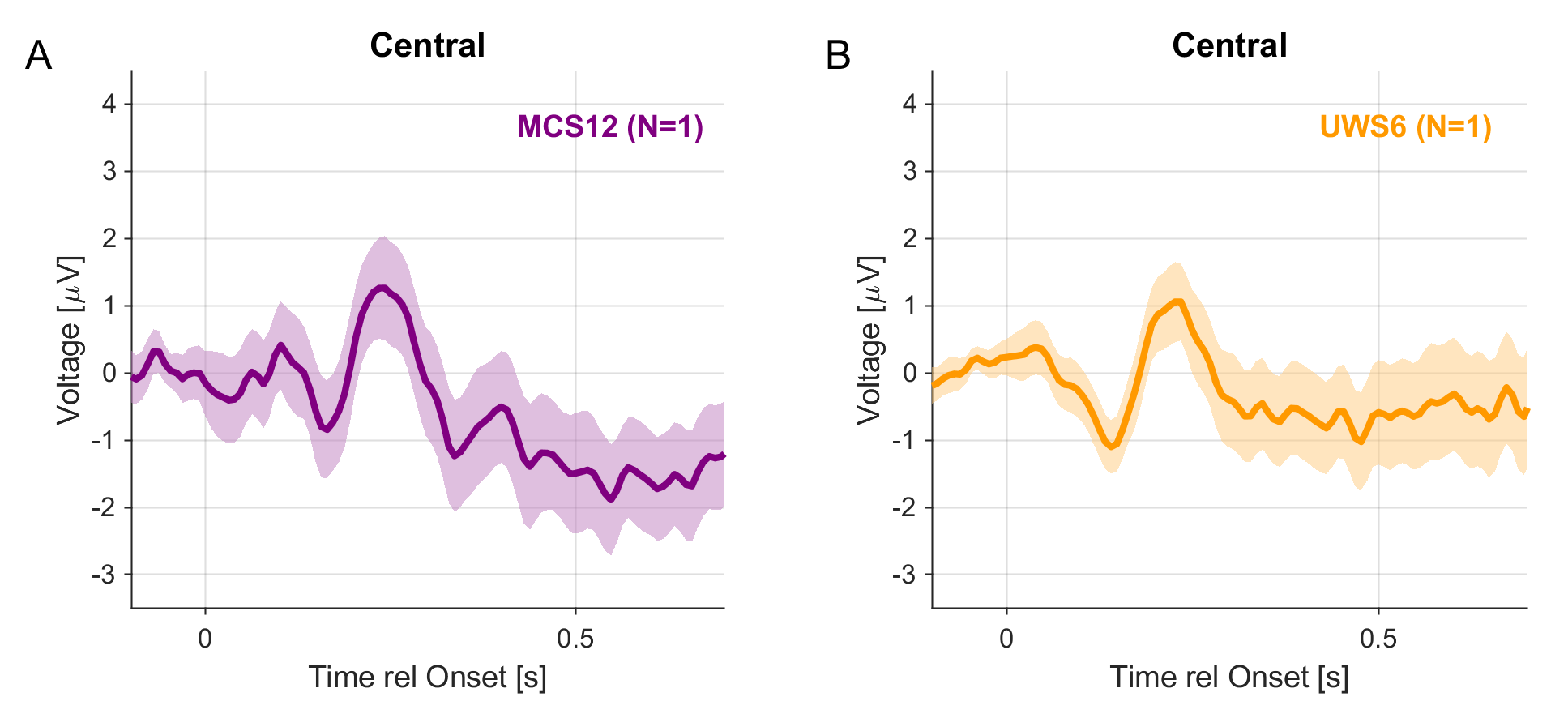
**Supplementary Figure 1.** Onset response for selected patients.

**A** a minimally conscious state patient (MCS12)

**B** unresponsive wakefulness syndrome (UWS6).

See Supplementary Table 1 for amplitude and latency values of P2 response, respectively), indicating that a clear onset response is present in a subset of PDOC patients.


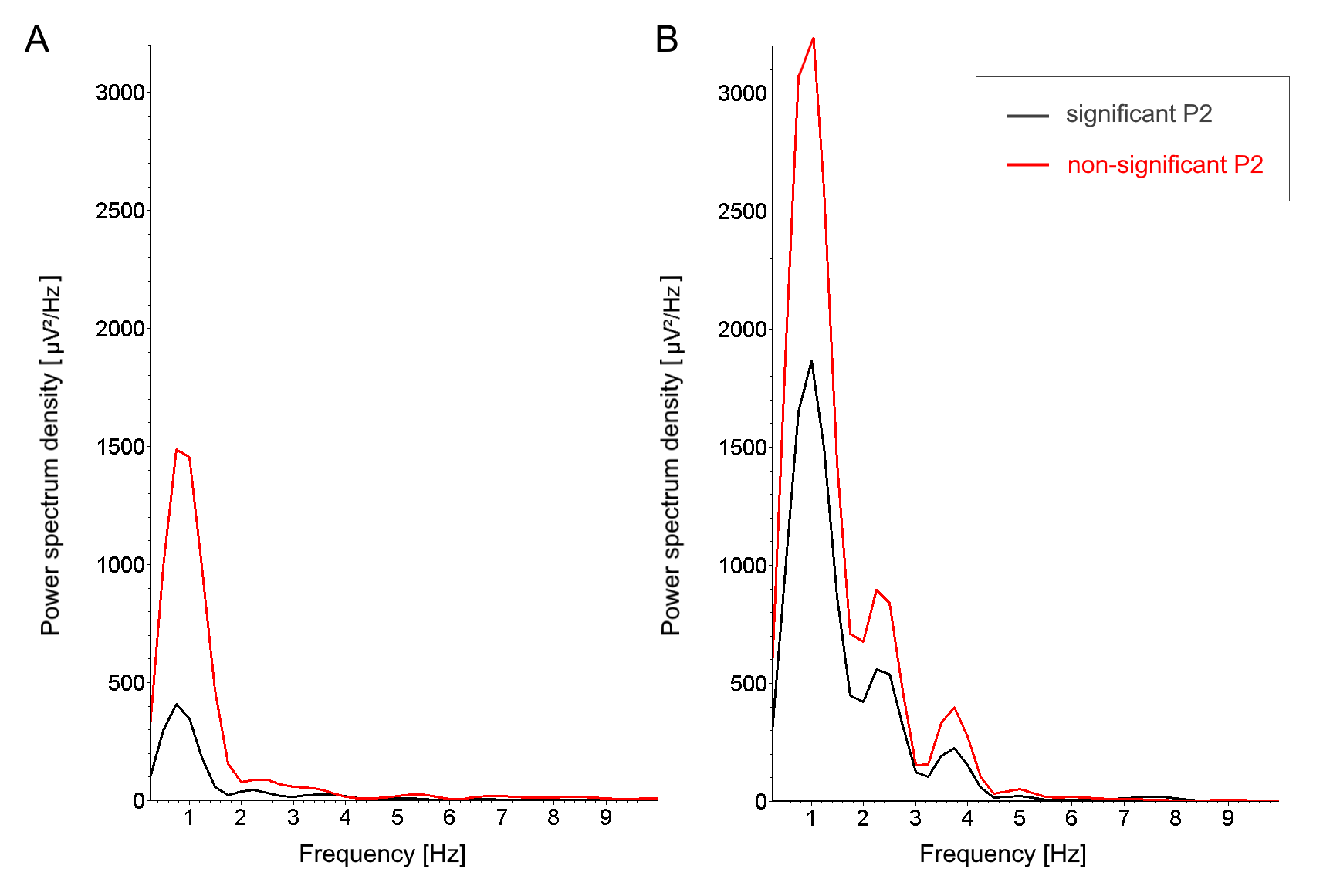


**Supplementary Figure 2:** Average spectrum for (**A**) a MCS and (**B**) an UWS patient, both divided with respect to the significant or non-significant P2 potential.

**
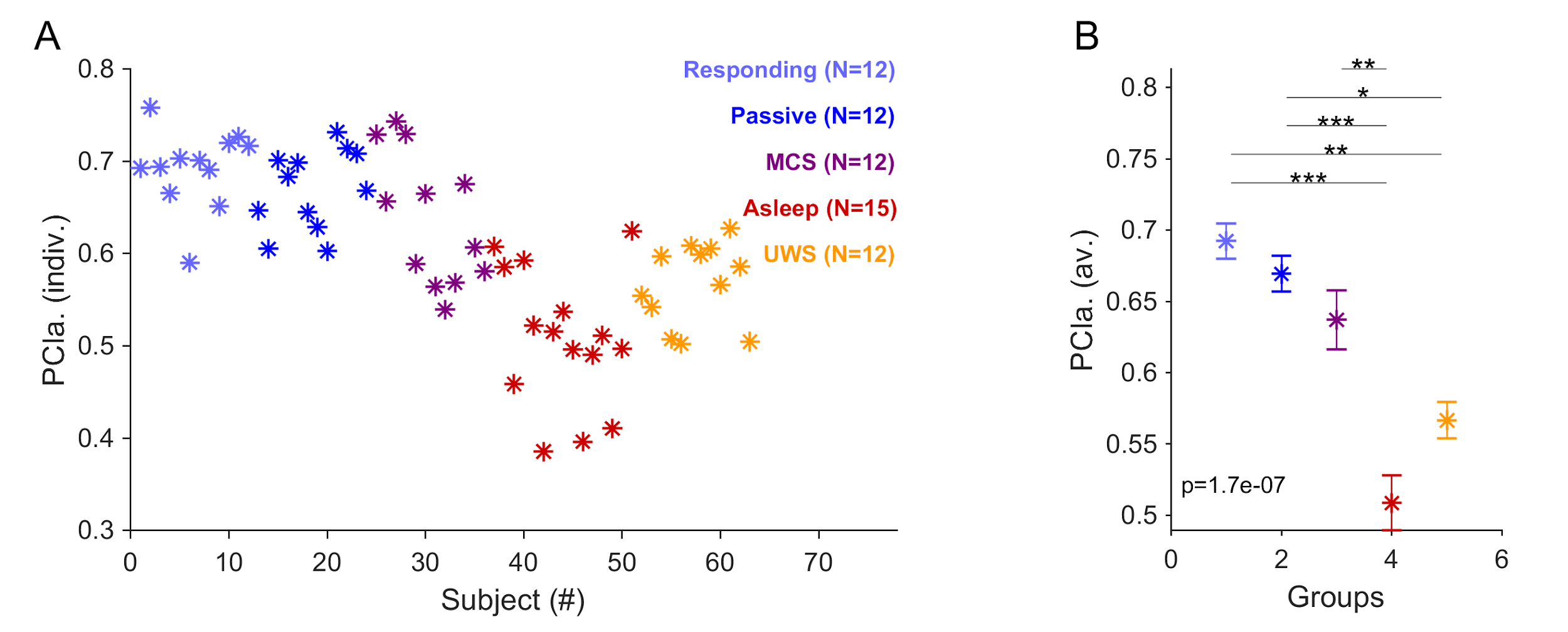
**

**Supplementary Figure 3:** PCIa around the stimulus onset differentiates less significantly between conscious and unconscious groups than at change time.

**A** PCIa values computed in a time window of [-0.25,1.25] s around the onset exhibited similar variability and range, but less pronounced differences between the groups.

**B** The PCIa scores across all groups were non-significant as were the post-hoc tests, with the averages exhibiting an inconsistent pattern with low effect sizes.


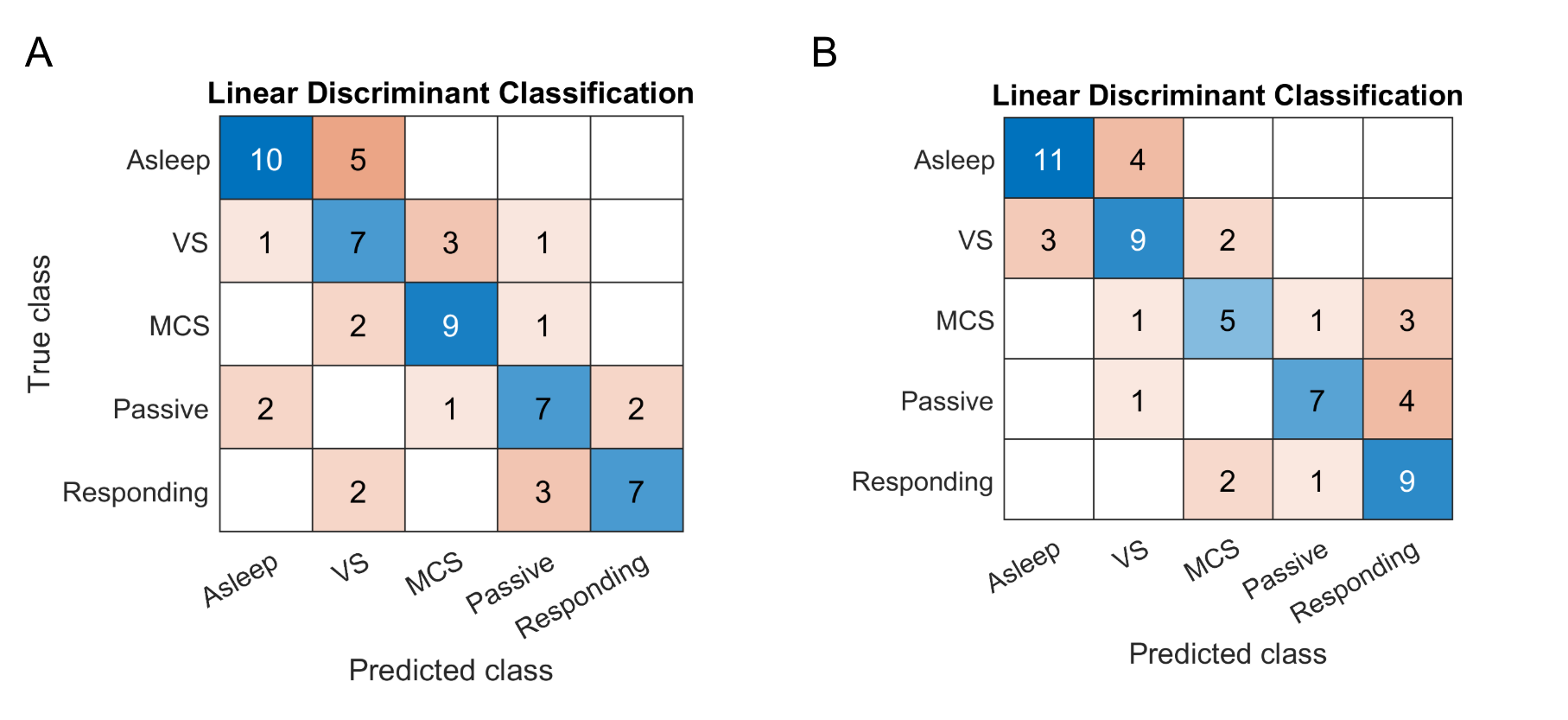


**Supplementary Figure 4:** Linear discriminant classification across all groups

**A** when the classification into MCS and UWS is based on the CRS-R measurements closest to the experiment,

**B** when it was based on the latest CRS-R measurement available.

They both indicate that there is significant identifiability of all groups, and confusions (off-diagonal classifications) are more frequent in the purportedly conscious and unconscious groups, respectively.
